# On the Treatment of Carbuncle with Escharotics

**Published:** 1822-09

**Authors:** Robert Swallow

**Affiliations:** Staff Surgeon.


### Art. VI.—*On the Treatment of Carbuncle with Escharotics.* By Robert Swallow, Staff Surgeon.

As I believe the following mode of treating that painful and distressing disease, carbuncle, is not generally known, I take the liberty of trespassing on the pages of your valuable Journal to insert two cases successfully treated by the remedy recommended by Mr. Blackadder, in his excellent treatise on Hospital Gangrene.

Lord Clermont, who requested me to publish his case, had been attacked with the above disease in London, and confined to his room nearly two months, under the care of my friend Mr. Guthrie. His lordship was cautioned by him to apply immediately for medical advice, if he should be attacked a second time. His lordship had been only a short time in the country before he felt an uneasiness and pain at the back part of the neck on the cervical vertebræ, which rapidly increased. Being at a friend's house, he did not send for me until the third day after perceiving the disease. On my being consulted, I found it to be a true characterized carbuncle; and, as he had just recovered from a long confinement from a similar disease, I advised his lordship to submit to its being destroyed by an escharotic, to which he readily consented. I made, therefore, a crucial incision, the full depth, length, and breadth of the disease, and filled up the incised places with dossils of lint well moistened with equal parts of liq. arsen. and water: the latter was renewed every hour or two. After twenty-four hours' application, a slight eschar began to form, the surrounding inflammation and pain evidently diminishing. Continuing it twelve hours more, a sufficient eschar was formed, and the pain and inflammation ceased. A common poultice was then applied very frequently, until the eschar separated, which, leaving a clean wound, was healed with the common simple dressings.

The other case of the disease occurred to a poor woman in this town; was situated in the dorsal vertebræ, and had existed ten days before she applied for advice: it was, therefore, of considerable size, and in a state of ulceration. Similar incisions were made as in his lordship's case, and the same remedy applied with the like beneficial effects: the pain and peculiar inflammation attending the disease subsiding as soon as the eschar began to form.

Some of my medical brethren may probably be afraid of absorption of the liq. arsen. producing baneful effects: but, in the many cases I have seen of hospital gangrene, (and I had charge of three large hospitals in Spain where only cases of that disease were admitted,) I never saw any unpleasant symptoms from the above cause; the quick formation of the eschar, I suppose, preventing the absorption, which shows the necessity of renewing the application every hour or two to produce that effect. No medicines of any consequence were used in either case.

*Watton, Norfolk; July 4th,* 1822.

